# Safety and efficacy of zero-fluoroscopy catheter ablation for paroxysmal supraventricular tachycardia in Chinese children

**DOI:** 10.3389/fcvm.2022.979577

**Published:** 2022-09-09

**Authors:** Xiaoran Cui, Ruibin Li, Wenli Zhou, Xiaohui Zhang, Xiaoning Wang, Jidong Zhang

**Affiliations:** ^1^Department of Cardiology, The Second Hospital of Hebei Medical University, Shijiazhuang, China; ^2^Department of Pediatric Cardiology, The Second Hospital of Hebei Medical University, Shijiazhuang, China

**Keywords:** Chinese children, paroxysmal supraventricular tachycardia, radiofrequency catheter ablation, zero-fluoroscopy, CARTO 3 system

## Abstract

**Objectives:**

To compare the safety and efficacy of completely zero-fluoroscopy radiofrequency ablation (RFA) with that of conventional RFA guided by three-dimensional mapping in Chinese children with paroxysmal supraventricular tachycardia (PSVT).

**Methods:**

The study had a single-center observational design and included 46 children aged 6–14 years who underwent RFA for PSVT at the Second Hospital of Hebei Medical University between March 2019 and September 2021. The children were divided according to whether they underwent zero-fluoroscopy RFA (zero-fluoroscopy group, *n* = 26) or routine RFA under X-ray guidance (conventional group, *n* = 20). Three-dimensional mapping was used in both groups. Baseline characteristics, total procedure time, RFA time, volume and duration of X-ray exposure, target mapping time, the immediate RFA success rate, incidence of complications, and recurrence rate were compared between the two groups.

**Results:**

The children had a median age of 12 years (interquartile range 10, 13), 47.8% (22/46) were boys, and 52.2% (24/46) were girls. The mean body weight was 48.75 ± 15.26 kg. There was no significant between-group difference in the baseline data (*P* > 0.05). All children were followed up as outpatients at 1, 3, and 6 months postoperatively. The target mapping time was significantly longer in the zero-fluoroscopy group than in the conventional group (12.96 ± 2.24 min vs. 6.65 ± 2.56 min, *P* < 0.05); however, there was no significant between-group difference in the immediate success rate (100% vs. 100%), success rate at 6 months postoperatively (92.30% vs. 95.00%), complication rate (0% vs. 0.05%), recurrence rate (7.70% vs. 5.00%), RFA time (212.50 s vs. 214.00 s), or total procedure time (78.50 min vs. 74.00 min) (*P* > 0.05).

**Conclusion:**

Zero-fluoroscopy catheter ablation can completely avoid fluoroscopy exposure in children without affecting the safety and efficacy of RFA.

## Introduction

Paroxysmal supraventricular tachycardia (PSVT) is an umbrella term used to describe a rapid heart rhythm that originates above the ventricles or involves a route not limited to the ventricles. PSVT is the most common symptomatic tachycardia in children (1), with an incidence of 0.1–0.4% (2). Atrioventricular nodal reentrant tachycardia (AVNRT), atrioventricular reentrant tachycardia (AVRT), and atrial tachycardia (AT) are the most common types of supraventricular tachycardia (SVT) in children (3, 4). Tachycardia that is present after the age of 5 years persists in more than 75% of cases (5). Treatment for PSVT includes physical stimulation, pharmacologic cardioversion, electrochemical therapy, and radiofrequency catheter ablation (RFCA) (6). However, none of these methods have a curative effect. Since the first report on RFCA as a therapy for tachyarrhythmia in children was published in 1991, RFCA has become the preferred curative strategy for most types of pediatric tachyarrhythmia (7). Several multicenter retrospective and prospective studies have reported that the success rate of RFCA in the treatment of tachyarrhythmia in children is comparable to that in adults (3, 8, 9) and that the children’s quality of life is enhanced (10). RFCA has been confirmed to be a safe and effective treatment for PSVT in children (11).

Nonetheless, fluoroscopy has potentially serious adverse effects on both patients and operators. Children are particularly at high risk of radiation-induced malignancy and other adverse effects because they are still developing and have a longer life expectancy than adults. The risk for a child younger than 14 years of age is approximately twice that of a 35-year-old adult (12). The principle of “as low as possible reasonably achievable” is recommended in children intraoperatively to limit radiation damage (13). The increasing familiarity with three-dimensional mapping (14, 15) and intracardiac echocardiography (ICE) (16–18) could reduce the fluoroscopy time. Zero or near-zero X-ray catheter ablation has been used to treat various types of arrhythmia and has been demonstrated to be safe and effective (19–21). However, few studies have investigated the efficacy and safety of completely zero-fluoroscopy ablation for PSVT in Chinese children.

After years of accumulated experience, we have mastered the ability to perform completely zero-fluoroscopy radiofrequency ablation (RFA). The purpose of this research was to compare the safety and efficacy of zero-fluoroscopy RFCA with that of conventional RFCA in the treatment of PSVT in Chinese children.

## Materials and methods

### Clinical information

**FIGURE 1:**
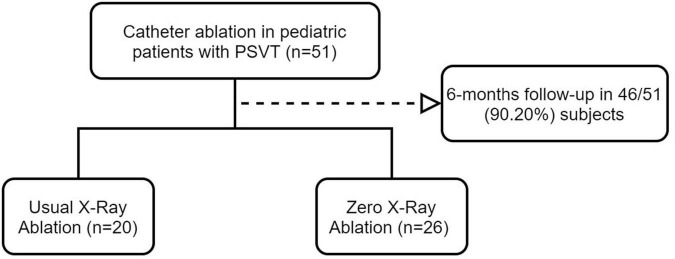
Flow chart showing the patient selection process.

Fifty-one children aged 6–14 years with PSVT treated with conventional or zero-fluoroscopy RFCA at the Second Hospital of Hebei Medical University between March 2019 and September 2021 were enrolled consecutively in this single-center observational study. Five patients were lost to follow-up, leaving 46 patients for inclusion in the study. Zero-fluoroscopy RFA was performed in 26 children (the zero-fluoroscopy group) and routine RFA was performed under radiographic guidance in 20 (the conventional group) (Figure 1). Total three-dimensional mapping was performed using the CARTO 3 System (Biosense Webster, Diamond Bar, CA, United States) in both groups. In accordance with the class I and IIa indications outlined in the “Expert consensus statement of China on the use of catheter ablation in children” (22), the following inclusion criteria were applied: (1) successful resuscitation after cardiac arrest due to pre-excitation syndrome; (2) accompanied by cardiac dysfunction or recurrent or persistent SVT; (3) symptomatic SVT with body weight ≥ 15 kg or pre-excitation cardiomyopathy not responsive to pharmacologic treatment; (4) SVT controllable by pharmacologic therapy and body weight ≥ 15 kg; (5) pharmacologic therapy for SVT ineffective or intolerable and body weight < 15 kg; (6) pre-excitation cardiomyopathy with body weight < 15 kg and pharmacotherapy ineffective; and (7) preoperative Ebstein’s anomaly with pre-excitation syndrome and body weight ≥ 15 kg.

Children were excluded if they had congenital heart disease, such as ventricular septal defect, atrial septal defect, or patent ductus arteriosus, if they had atrial fibrillation or ventricular arrhythmia, a bleeding tendency, or serious liver or kidney dysfunction, if they were unable to tolerate RFCA or had other contraindications, and if they were unable to attend for follow-up. The research was approved by the Ethics Committee of the Second Hospital of Hebei Medical University (approval number: 2022-R074). A signed informed consent form was obtained from a parent or guardian of each child before the ablation procedure.

### Preparation

All children were required to cease their antiarrhythmic medication for more than 5 half-lives before the procedure. The type of SVT and its mechanism were determined before the ablation procedure according to the characteristics seen on the electrocardiogram (ECG). Local anesthesia was used in older children who could cooperate with RFCA, and general anesthesia with endotracheal intubation and mechanical ventilation was used in younger children who could not cooperate. The body surface electrode was linked to an EP recorder and the CARTO 3 System in both study groups. If arrhythmia could not be induced in the resting state, an electrophysiology (EP) study was repeated after intravenous administration of isoproterenol to determine the type of arrhythmia.

### Ablation approach

#### Electrophysiology study

Programmed ventricular/atrial stimulation was applied, and an intracardiac EP study was performed to determine the type of SVT.

AVNRT was diagnosed based on the following: (1) evidence of AH/AV jumping; (2) a VA interval showing decreased conduction; (3) the A wave reversed at the bundle of His on the ECG; and (4) the H wave in front of the V wave at the time of SVT with an HA interval of 35–55 ms. Any two of these four conditions were required for a diagnosis of AVNRT.

AVRT was diagnosed if (1) the VA interval had no Wenckebach conduction and conduction was all or none, (2) the AH interval was within the normal range, and (3) eccentric atrio-atrial retrograde transmission could be diagnosed as an atrioventricular (AV) accessory pathway and the AV interval of centripetal ventriculoatrial conduction at onset was > 70 ms.

A diagnosis of AT was made if the EP study showed the following: (1) a normal AH interval; (2) a normal HV interval, AV < VA; (3) 1:1 AV conduction or Wenckebach block and AT still present under AV block; and (4) order of atrial activation different from that of a sinus rhythm.

### Zero-fluoroscopy group

**FIGURE 2:**
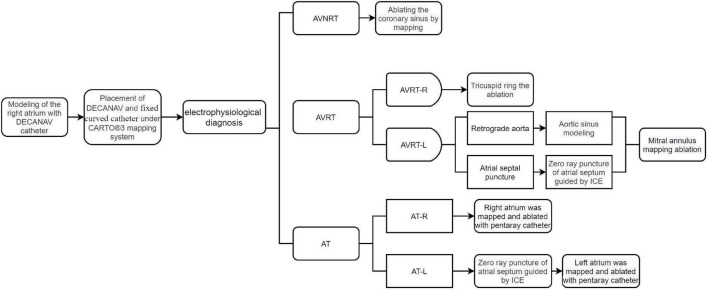
Diagram showing how zero-fluoroscopy RFCA was performed according to type of supraventricular tachycardia. AT, atrial tachycardia; AT-L, left-sided AT; AT-R, right-sided AT; AVNRT, atrioventricular nodal reentrant tachycardia; AVRT, atrioventricular reentrant tachycardia; AVRT-L, left-sided AVRT; AVRT-R, right-sided AVRT; ICE, intracardiac echocardiography.

In the zero-fluoroscopy group, RFCA was performed under the CARTO 3 system without X-ray fluoroscopy. The patient was placed in a supine position on the operating table, the inguinal area was routinely disinfected on both sides, surgical drapes were applied, 1% lidocaine was applied for local anesthesia, and the right femoral vein was punctured. A decapolar Decanav catheter (Biosense Webster) was advanced into the right atrium (RA) through the right femoral vein, and fast anatomic mapping of the inferior vena cava, coronary sinus, RA, and tricuspid valve was started using the catheter. The Decanav catheter was then placed in the coronary sinus, and a quadripolar catheter (Biosense Webster) was placed in the apex of the right ventricle for EP study. His catheter is also recommended during EP study when necessary. Figure 2 shows how zero-fluoroscopy RFCA was performed according to the SVT type.

#### Targets and procedures for zero-fluoroscopy radiofrequency catheter ablation

**FIGURE 3:**
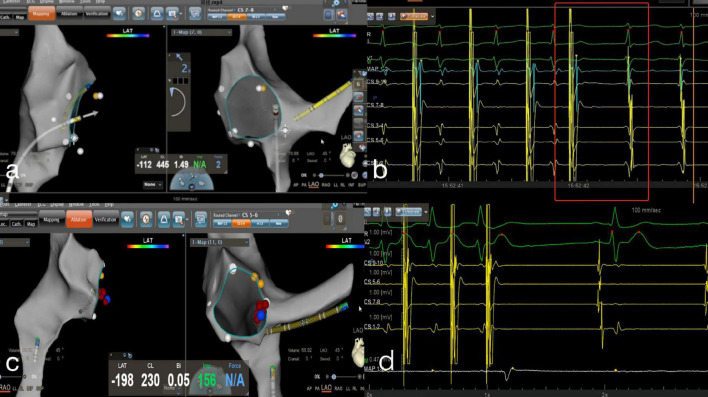
Zero-fluoroscopy radiofrequency ablation for AVNRT. **(a)** Modeling of the right atrium was performed with a Decanav catheter using CARTO 3 system. The Decanav catheter the apex of the right ventricle. **(b)** An electrophysiology study showed AH/AV jumping. The slow-fast type of AVNRT was diagnosed. **(c)** The level of the lower portion of the coronary sinus ostium was suggested to be the slow pathway location. **(d)** AV node jumping disappeared, and AVNRT was not induced. AV, atrioventricular; AVNRT, AVNRT, atrioventricular nodal reentrant tachycardia.

The ablation targets and procedures for AVNRT were as follows: (1) placement of test ablations low in the triangle of Koch (at the level of the lower portion of the coronary sinus ostium), with a wave variation in A or V of less than 10% or persistence of the dominant waveform; (2) no His signal; and (3) an A: V ratio between 1:2 and 1:10, which is typically chosen to mark an appropriate location within the triangle of Koch. Low-amplitude elongated atrial signals have been suggested to represent the slow pathway potential of the antegrade limb in the right inferior extension area. Temperature-controlled ablation with an energy of 20–35 W and a temperature of 50–55°C was set using a Navistar Inquiry steerable diagnostic catheter (Irvine Biomedical, St Paul, MN, United States) supported by an SL1 long sheath (Abbott Medical, Abbott Park, IL, United States). Decelerated junctional rhythms were observed within 10 s, and the impulse was applied for a period of 60–120 s. The change in rhythm was closely monitored during the ablation. If no decelerated junctional rhythms were observed, the catheter position was changed to the next location. Figure 3 illustrates the RFCA procedure for AVNRT in the zero-fluoroscopy group.

**FIGURE 4:**
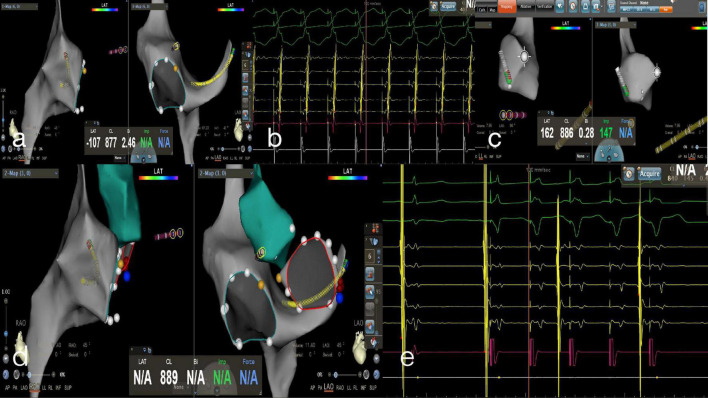
Zero-fluoroscopy radiofrequency ablation for AVRT. **(a)** Modeling of the right atrium was performed with a Decanav catheter using the CARTO 3 System. The Decanav catheter was placed in the coronary sinus and a quadripolar catheter was placed in the apex of the right ventricle. **(b)** During the onset of SVT stimulated by S1S1, the retrograde A wave showed eccentric conduction and was diagnosed as a left concealed accessory pathway. **(c)** The retrograde aortic method was used to deliver the ablation catheter to the aortic root and establish a model of the aorta. **(d)** The ablation catheter electrode crosses the aortic valve to model the mitral annulus, and the ablation target is located at three o’clock. **(e)** An electrophysiology study showed ventricular pacing with ventricular-atrial separation and retrograde transmission through atrioventricular node. The retrograde A wave was centripetal.

The following procedures were followed for AVRT. The ablation targets in an overt accessory pathway were those with the earliest ventricular activation (EVA) and/or the retrograde earliest atrial activation (EAA). The ablation target in a concealed accessory pathway was the retrograde EAA (i.e., local fusion of V and A waves, or at the time of no conduction delay in the accessory pathway, a local VA interval ≤ 40 ms). The EVA was more than 20 ms earlier than the earliest δ wave on the body surface at the start of the local V wave, the AV interval in the local electrogram ≤ 40 ms without conduction delay in the accessory pathway, or the A and V waves fused (equipotential line between A and V waves ≤ 5 ms). Ablation was performed in cold saline power mode with an energy of 30 W and a cut-off temperature of 45°C. The accessory pathway was blocked within 10 s, resulting in an impulse duration of 60–120 s. A left-sided accessory pathway required either a trans-septal approach under the guidance of ICE or a retrograde transaortic approach. The ablation target and ablation mode were confirmed in the same way as for the right accessory pathway. Figure 4 displays the RFCA procedure for AVRT under zero-fluoroscopy guidance.

For right AT, a star-shaped PentaRay mapping catheter (Biosense Webster) was advanced into the RA *via* the femoral vein. The origin of the AT was determined by a combination of activation mapping and entrainment mapping. The ablation target in macroreentrant AT is usually the critical isthmus. The ablation target in focal AT is EAA, and activation mapping is most often used. The EAA shows negative and positive bidirectional waves in the bipolar recording. During unipolar mapping, the target recording shows a QS pattern. A ThermoCool SmartTouch catheter (Biosense Webster) was used for ablation. During the ablation, the cold saline power mode was used at an ablation energy of 35 W and a cut-off temperature of 45^°^C. If a sinus rhythm was achieved after a period of 10 s, an impulse duration of 60–120 s was performed, and the target was remapped if not.

Left AT was located primarily around the pulmonary vein or in the left atrial auricle. The PentaRay catheter was advanced into the left atrium for modeling and mapping utilizing a trans-septal puncture technique under the guidance of ICE. An ablation catheter was used for the ablation procedure, and the target mapping and ablation modes were the same as those used for right AT.

### Conventional group

The CARTO 3 System combined with X-ray fluoroscopy was used in the conventional group. The patient preparation was the same as in the zero-fluoroscopy group. A Decanav catheter was placed into the coronary sinus and a quadripolar catheter into the apex of the right ventricle under fluoroscopic guidance. The type of SVT was determined by an intracardiac EP study. The CARTO 3 System combined with X-ray fluoroscopy was used for target mapping and location, and the standard target location was the same as that in the zero-fluoroscopy group.

#### Procedure

For AVNRT, a Navistar catheter was advanced into the RA *via* the right femoral vein under fluoroscopy to model the RA and tricuspid valve geometry. RFCA was performed at the ablation target, and the target location was consistent with that in the zero-fluoroscopy group.

For AVRT, a Navistar catheter was placed *via* a right accessory pathway and the modeling was performed in the same way as for AVNRT. For the left accessory pathway, the operator selected a retrograde aortic approach or trans-septal puncture into the left ventricle, hooking the mitral annulus. RFA was performed at the ablation target, and the target location and ablation mode were the same as in the zero-fluoroscopy group.

For right AT, after the catheter was placed under X-ray guidance, the rest of the procedure was the same as in the zero-fluoroscopy group. Inter-atrial septal puncture was performed for left AT under fluoroscopy. The PentaRay was delivered to the left atrium for modeling and mapping. An ablation catheter was applied, and the target location, ablation mode, and ablation parameters were the same as in the zero-fluoroscopy group.

#### Ablation endpoints

RFCA was considered effective for AVNRT after observation for 30 min if atrial and ventricular EP stimulation did not induce AVNRT, the slow pathway conduction disappeared, or residual slow pathway conduction was unaccompanied or only accompanied by a single atrial echo.

RFCA was deemed to be successful for AVRT after observation for 30 min if the ECG and pacemaker mapping confirmed blocking of the functions of sequential/reverse transmission of the accessory pathway, disappearance of the pre-excitation pattern on the surface ECG, and isolation of ventricular pacing or reversal at the AV node.

AT was deemed to be treated successfully by RFCA after observation for 30 min if isoproterenol and atrial stimulation did not induce AT.

#### Follow-up

Routine and comprehensive ECG monitoring was performed immediately after the patient returned to the ward. Routine follow-up was performed at 1, 3, and 6 months in the outpatient department. During the follow-up, transesophageal atrial pacing could be performed for patients with frequent symptoms but a normal dynamic ECG to check for recurrence of PSVT. For patients with recurrence, the decision to perform an EP study and RFCA was determined by the family.

### Statistical analysis

Data that were distributed normally are expressed as the mean ± standard deviation and those that were not distributed normally are expressed as the median (interquartile range). Variables were compared between groups using the *t*-test or rank-sum test. Qualitative data were examined using the chi-squared test or Fisher’s exact test. All data were analyzed using SPSS version 23.0 software (IBM Corp., Armonk, NY, United States). A *p*-value < 0.05 was considered statistically significant.

## Results

The children had a median age of 12 years (IQR, 10, 13), 47.8% (22/46) were boys, and 52.2% (24/46) were girls. The average height was 157.46 ± 14.20 cm, and the mean weight was 48.75 ± 15.26 kg. There was no significant between-group difference in body mass index, age, sex, height, weight, disease course, left atrial diameter, left ventricular end-diastolic diameter, right atrial diameter, right ventricular diameter, or left ventricular ejection fraction between the zero-fluoroscopy group and the conventional group (*P* > 0.05). The baseline data are shown in Table 1.

**TABLE 1:** Patient demographics and clinical characteristics according to whether conventional or zero-fluoroscopy radiofrequency cardiac ablation was performed.

Item	Total population	Zero radiofrequency group	Conventional group	*P*
Case number	46	26	20	–
Male (*n*, %)	22 (47.8%)	12 (46.2)	10 (50.0)	0.796
Age (year-old)	12 (10, 13)	12 (10, 13)	12 (10, 14)	0.686
Height (cm)	157.46 ± 14.20	156.92 ± 13.39	158.15 ± 15.51	0.775
Body weight (kg)	48.75 ± 15.26	48.65 ± 14.62	48.87 ± 16.43	0.962
BMI	18.84 (15.96, 22.16)	19.04 (15.96, 21.98)	18.52 (16.05, 22.38)	0.859
Disease course (months)	12.00 (3.00, 24.00)	12.00 (3.75, 27.00)	10.50 (2.25, 22.75)	0.474
Left atrial diameter (mm)	25.28 ± 4.37	25.00 ± 3.86	25.65 ± 4.05	0.623
Left ventricular end-diastolic diameter (mm)	40.00 (36.75, 44.00)	42.00 (38.75, 44.00)	39.00 (31.50, 43.75)	0.234
Right atrial diameter (mm)	29.00 (25.00, 31.00)	29.50 (25.75, 31.00)	29.00 (25.00, 31.00)	0.509
Right ventricular diameter (mm)	15.74 ± 2.45	16.04 ± 2.34	15.35 ± 2.60	0.351
Left ventricular ejection fraction	65.00 (62.97, 69.23)	66.20 (62.50, 70.88)	64.70 (63.25, 66.43)	0.191

BMI, body mass index.

Of the 46 children treated by RFCA, 18 had AVNRT (58.7%), 27 had AVRT (39.13%), and 1 had AT (2.1%). AVRT was dominant in PSVT, accounting for 58.70% of cases (Table 1); AVNRT was mainly the slow-fast type (83.33%). AVRT was found predominantly in the left accessory pathway (55.56%).

There was no significant difference in the proportions of patients in the conventional group and zero-fluoroscopy group with AVNRT (8 vs. 10) or AVRT (17 vs. 10). There was also no significant between-group difference in the types of PSVT (*P* > 0.05). All AVNRT was of the slow-fast type in the zero-fluoroscopy group; however, in the conventional group, seven were the slow-fast type, one was the fast-slow type, and two were the slow-slow type. In the zero-fluoroscopy group and conventional group, there were, respectively, eight and four cases of AVRT in the right accessory pathway (including four and one in a right concealed accessory pathway and four and three in an overt accessory pathway) and nine and six cases in a left accessory pathway (including six and four in a left concealed right accessory pathway and three and two in a left overt accessory pathway). There was one case of AT in the zero-fluoroscopy group and none in the conventional group (Table 2).

**TABLE 2:** Types of supraventricular tachycardia.

Types of ventricular tachycardia	Total population	Zero radiofrequency group	Conventional group
Slow-fast	15	8	7
Fast-slow (*n*)	1	0	1
Slow-slow (*n*)	2	0	2
AVRT (*n*, %)	27 (58.70)	17 (65.4)	10 (50.0)*
Right accessory pathway (*n*)	12	8	4
Concealed accessory pathway (*n*)	5	4	1
Dominant accessory pathway (*n*)	7	4	3
Left accessory pathway (*n*)	15	9	6
Concealed accessory pathway (*n*)	10	6	4
overt accessory pathway (*n*)	5	3	2
AT (*n*, %)	1 (2.17)	1 (3.8)	0 (0)

*P < 0.05 vs. zero-fluoroscopy group. AT, atrial tachycardia; AVRT, atrioventricular reentrant tachycardia.

The target mapping time was significantly longer in the zero-fluoroscopy group than in the conventional group (12.96 ± 2.24 min vs. 6.65 ± 2.56 min, *P* < 0.05); however, there was no significant between-group difference in the immediate success rate (100% vs. 100%), 6-month follow-up success rate (92.30% vs. 95.00%), incidence of complications (0% vs. 0.05%), recurrence rate (7.70% vs. 5.00%), RFA time (212.50 s vs. 214.00 s), or total procedure time (78.50 min vs.74.00 min) (*P* > 0.05). The exposure dose and exposure time were 5.40 ± 1.82 mGy and 2.95 ± 0.82 min, respectively, in the conventional group, and both these values were zero in the zero-fluoroscopy group; the between-group difference was statistically significant (*P* < 0.05). There was no significant between-group difference in the incidence of complications (*P* > 0.05). There were no complications in the zero-fluoroscopy group and 1 case of arteriovenous fistula on the second postoperative day in the conventional group. At the 6-month follow-up, there was no statistically significant difference in the recurrence rate between the zero-fluoroscopy group and the conventional group (7.70% vs. 5.00%, *P* > 0.05). Two patients in the zero-fluoroscopy group underwent a repeat RFCA at our hospital, one for slow-fast AVNRT and the other for AVRT with a right-sided overt accessory pathway. In the conventional group, one patient had a recurrence of slow-slow AVNRT and underwent repeat RFCA in a hospital in Beijing. None of the patients who underwent a second RFCA procedure had a recurrence during the follow-up (Table 3).

**TABLE 3:** Radiofrequency cardiac ablation parameters and follow-up results.

Item	Zero radiofrequency group	Conventional group	*P*
Total procedure time (min)	78.50 (60.00, 106.25)	74.00 (60.00, 91.50)	0.452
Target mapping time (min)	12.96 ± 2.24	6.65 ± 2.56	0.000
Exposure dose (mGy)	0	5.40 ± 1.82	0.000
Exposure time (min)	0	2.95 ± 0.82	0.000
RFA time (s)	212.50 (190.75, 240.00)	214.00 (194.50, 250.75)	0.485
Immediate success rate (%)	100	100	1.000
6-month success rate (%)	92.30	95.00	1.000
Complications (case)	0	1	0.435
Recurrence rate (%)	7.70	5.00	1.000

RAF, radiofrequency ablation.

## Discussion

The efficacy and benefits of RFCA for SVT in children have been well demonstrated (4, 23, 24). RFCA can be used as an effective alternative to medical therapy in children older than 5 years of age and is recommended when medical therapy is either not effective or associated with intolerable adverse effects in those under 5 years of age, including infants. Children weighing < 15 kg or aged younger than 5 years have been shown to be at increased risk of complications during RFA, and catheter ablation should be limited to patients with clear indications (4). With the developments in technology, several new techniques, including ICE, MRI-guided cardiac ablation, and three-dimensional mapping, have been used to reduce exposure to radiation in the clinical setting (25, 26). Three of these three-dimensional mapping systems are now in widespread clinical use, namely, the CARTO 3 system, the EnSite NavX system (Abbott/St. Jude Medical, Inc., St. Paul, MN, United States), and the Rhythmia HDx system (Boston Scientific Inc., Marlborough, MA, United States). The CARTO system is more precise for the location of the catheter tip than the EnSite NavX system and has a pressure sensing module compared to the Rhythmia system. At the same time, it has the function of real-time ultrasound integration. Intracardiac cardiac ultrasonography using ICE is now generally available. ICE can more precisely delineate cardiac structures and allows for visualizing catheter contact and transmural lesion formation in real time. The combination of ICE and the CARTO 3 System increases the safety and effectiveness of trans-septal puncture during ablation procedures (27, 28). Nevertheless, in this study, left-sided tachycardia could be treated successfully *via* a retrograde transaortic approach without the use of ICE, thereby reducing the cost to the patient.

In this research, although the duration of electrode placement was longer in the zero-fluoroscopy group, the immediate success rate was 100% in both groups. At the end of 6 months of follow-up, the success rates for AVRT, AVNRT, and AT were 97.8, 95.7, and 100%, respectively. The procedural success rate was 92.3% in the zero-fluoroscopy group and 95% in the conventional group, with an overall success rate of 93.7%, which is similar to that in a previous study (29). Nevertheless, owing to the different age composition of the children, the ablation success rate for the different types of SVT is slightly different from that in previous studies. In our study, there were no statistically significant differences between the two groups in terms of overall procedural success rates or recurrence rates. Although the target mapping time was prolonged in the zero-fluoroscopy group, zero-fluoroscopy RFCA did not increase the total procedural time, indicating that this type of ablation is clinically effective for SVT in children.

The risks of RFCA in children are similar to those in adults and include AV block, bleeding, infection, arteriovenous fistula, cardiac perforation, valve injury, thrombosis or embolus formation, coronary spasm, and radiation. Of these, AV block, cardiac perforation, and thrombosis or embolus formation are serious complications requiring urgent treatment (8). In this study, there was no significant difference in the incidence of complications between the groups, and no serious complications such as AV block, cardiac tamponade, thrombosis, or embolus formation in either group. An arteriovenous fistula occurred in one child in the conventional group postoperatively but settled after compression dressing. Therefore, RFCA using the CARTO 3 System can be considered safe for children (30, 31). Other researchers have confirmed the long-term safety of zero-fluoroscopy RFCA (32).

X-ray radiation is inevitable in conventional RFCA, and radiation exposure poses some degree of long-term risk to both patients and operators (33), especially growing children (34). Moreover, there have been a number of cases of left-sided brain and neck tumors in physicians performing interventions requiring radiation exposure over a long period of time (35). In our study, the zero-fluoroscopy group had no radiation exposure during the entire ablation procedure or any loss of procedural efficacy or safety. Zero-fluoroscopy RFCA can completely avoid fluoroscopy exposure during ablation, thereby reducing the risk of malignancy in children (36, 37). With the development of technology, people have an increasing need to lead a healthy life. Therefore, it is necessary to reduce exposure to X-ray fluoroscopy during RFCA. Both patients and operators will benefit from zero-fluoroscopy RFCA in the future. This procedure should be promoted and applied, especially in children who are more susceptible to radiation injury.

Zero-fluoroscopy RFCA under the CARTO 3 System has the following advantages: (1) when using a Decanav catheter, even if SVT cannot be induced during the procedure, the modeling can still be successfully completed successfully without an ablation catheter, which reduces the cost to patients, and (2) when a child’s lower limb vessels are tortuous, a long wire can be used if the catheter cannot be delivered smoothly. When the upper limb vessels require puncture, the internal jugular vein should be punctured where possible to reduce the need for surgical treatment. However, children have a tiny heart, a thin heart wall, small coronary sinus, and high requirements for electrode placement and discharge, so the procedure must be performed gently to avoid unnecessary injury. The time and energy of discharge ablation should be shorter and lower than that used in adults, and the catheter contact force should be maintained stably between 5 and 15 g.

## Limitations

This research has some limitations. First, the research population was small because of the limited number of children who required RFCA for PSVT. Further studies in larger sample sizes with longer follow-up periods are required to evaluate the long-term prognosis. Second, the research was limited to a population in China. Therefore, larger clinical studies are needed to investigate the safety and effectiveness of zero-fluoroscopy RFCA in children of other ethnicities.

## Conclusion

Zero-fluoroscopy RFCA with the CARTO 3 System is safe and effective in the treatment of PSVT in Chinese children aged 6–14 years and can completely avoid radiation exposure for both children and operators. PSVT in Chinese children can be treated by zero-fluoroscopy RFCA if conditions permit.

## Data availability statement

The raw data supporting the conclusions of this article will be made available by the authors, without undue reservation.

## Ethics statement

The studies involving human participants were reviewed and approved by the Second Hospital of Hebei Medical University (approval number: 2022-R074). Written informed consent to participate in this study was provided by the participants’ legal guardian/next of kin.

## Author contributions

XC wrote the original draft and contributed to the data collection. RL and WZ performed the statistical analysis. XZ and XW followed up the patients. JZ designed the study and revised the manuscript. All authors made editorial changes to the manuscript, read, and approved the final manuscript.

## Abbreviations

PSVT: paroxysmal supraventricular tachycardia
AVNRT: atrioventricular nodal reentrant tachycardia
AVRT: atrioventricular reentrant tachycardia
AT: atrial tachycardia
TK: triangle of Koch
RIE: right inferior extension
FAM: fast anatomic mapping
AV: atrioventricular
RFA: radiofrequency ablation
RFCA: radiofrequency catheter ablation
ICE: intracardiac echocardiography.

## References

[1] Paul T Bertram H Bökenkamp R Hausdorf G. Supraventricular tachycardia in infants, children and adolescents: diagnosis, and pharmacological and interventional therapy. *Paediatr Drugs.* (2000) 2:171–81. 10.2165/00128072-200002030-00002 10937468

[2] Calabrò MP Cerrito M Luzza F Oreto G. Supraventricular tachycardia in infants: epidemiology and clinical management. *Curr Pharm Des.* (2008) 14:723–8. 10.2174/138161208784007761 18393870

[3] Li X Li F Zeng S Yuan Y Guo B Han B Pediatric intra-cardiac electrophysiological study and radiofrequency catheter ablation of tachyarrhythmia——a national multicenter clinical study. *Chin J Cardiac Arrhythm.* (2014) 18:9–16.

[4] Philip Saul J Kanter RJ Abrams D Asirvatham S Bar-Cohen Y Blaufox AD PACES/HRS expert consensus statement on the use of catheter ablation in children and patients with congenital heart disease: developed in partnership with the pediatric and congenital electrophysiology society (PACES) and the heart rhythm society (HRS). Endorsed by the governing bodies of PACES, HRS, the American academy of pediatrics (AAP), the American heart association (AHA), and the association for European pediatric and congenital cardiology (AEPC). *Heart Rhythm.* (2016) 13:e251–89. 10.1016/j.hrthm.2016.02.009 26899545

[5] Perry JC Garson A Jr. Supraventricular tachycardia due to Wolff-Parkinson-White syndrome in children: early disappearance and late recurrence. *J Am Coll Cardiol.* (1990) 16:1215–20. 10.1016/0735-1097(90)90555-4 2229769

[6] Kugler JD Danford DA. Management of infants, children, and adolescents with paroxysmal supraventricular tachycardia. *J Pediatr.* (1996) 129:324–38. 10.1016/S0022-3476(96)70063-X 8804320

[7] Walsh EP Saul JP. Transcatheter ablation for pediatric tachyarrhythmias using radiofrequency electrical energy. *Pediatr Ann.* (1991) 20:386, 388–92. 10.3928/0090-4481-19910701-12 1923646

[8] Kugler JD Danford DA Houston KA Felix G. Pediatric radiofrequency catheter ablation registry success, fluoroscopy time, and complication rate for supraventricular tachycardia: comparison of early and recent eras. *J Cardiovasc Electrophysiol.* (2002) 13:336–41. 10.1046/j.1540-8167.2002.00336.x 12033349

[9] Van Hare GF Javitz H Carmelli D Saul JP Tanel RE Fischbach PS Prospective assessment after pediatric cardiac ablation: recurrence at 1 year after initially successful ablation of supraventricular tachycardia. *Heart Rhythm.* (2004) 1:188–96. 10.1016/j.hrthm.2004.03.067 15851152 PMC1892227

[10] Szafran E Baszko A Bukowska-Posadzy A Łaźniak A Moszura T Siwińska A Influence of ablation therapy on the quality of life in children with supraventricular tachycardia. *Eur Rev Med Pharmacol Sci.* (2017) 21:2550–9. 28617528

[11] Krause U Paul T Bella PD Gulletta S Gebauer RA Paech C Pediatric catheter ablation at the beginning of the 21st century: results from the European multicenter pediatric catheter ablation registry ‘EUROPA’. *Europace.* (2021) 23:431–40. 10.1093/europace/euaa325 33227133

[12] Shoei SM John MM. *Catheter Ablation of Cardiac Arrhythmias.* 4th ed. Philadelphia, PA: Elsevier Press (2020).

[13] Fazel R Gerber TC Balter S Brenner DJ Carr JJ Cerqueira MD Approaches to enhancing radiation safety in cardiovascular imaging: a scientific statement from the American heart association. *Circulation.* (2014) 130:1730–48. 10.1161/CIR.0000000000000048 25366837

[14] Knackstedt C Schauerte P Kirchhof P. Electro-anatomic mapping systems in arrhythmias. *Europace.* (2008) 10(Suppl. 3):iii28–34. 10.1093/europace/eun225 18955396

[15] Miyazaki A Blaufox AD Fairbrother DL Saul JP. Cryo-ablation for septal tachycardia substrates in pediatric patients: mid-term results. *J Am Coll Cardiol.* (2005) 45:581–8. 10.1016/j.jacc.2004.10.051 15708707

[16] Seizer P Bucher V Frische C Heinzmann D Gramlich M Müller I Efficacy and safety of zero-fluoroscopy ablation for supraventricular tachycardias. Use of optional contact force measurement for zero-fluoroscopy ablation in a clinical routine setting. *Herz.* (2016) 41:241–5. 10.1007/s00059-015-4358-4 26462477

[17] Ferguson JD Helms A Mangrum JM Mahapatra S Mason P Bilchick K Catheter ablation of atrial fibrillation without fluoroscopy using intracardiac echocardiography and electroanatomic mapping. *Circ Arrhythm Electrophysiol.* (2009) 2:611–9. 10.1161/CIRCEP.109.872093 20009075 PMC4570247

[18] Kerst G Weig HJ Weretka S Seizer P Hofbeck M Gawaz M Contact force-controlled zero-fluoroscopy catheter ablation of right-sided and left atrial arrhythmia substrates. *Heart Rhythm.* (2012) 9:709–14. 10.1016/j.hrthm.2011.12.025 22222276

[19] Wang Y Chen GZ Yao Y Bai Y Chu HM Ma KZ Ablation of idiopathic ventricular arrhythmia using zero-fluoroscopy approach with equivalent efficacy and less fatigue: a multicenter comparative study. *Medicine (Baltimore).* (2017) 96:e6080. 10.1097/MD.0000000000006080 28178165 PMC5313022

[20] Haegeli LM Stutz L Mohsen M Wolber T Brunckhorst C On CJ Feasibility of zero or near zero fluoroscopy during catheter ablation procedures. *Cardiol J.* (2019) 26:226–32. 10.5603/CJ.a2018.0029 29611170 PMC8086663

[21] Morka A Śledź J Deutsch K Ludwik B Zagrodzka M Szydłowski L Feasibility and performance of catheter ablation with zero-fluoroscopy approach for regular supraventricular tachycardia in patients with structural and/or congenital heart disease. *Medicine (Baltimore).* (2019) 98:e17333. 10.1097/MD.0000000000017333 31593082 PMC6799864

[22] Counseling and Electrophysiology Branch of Chinese Medical Association, Cardiovascular group of pediatric branch, Cardiovascular Professional Committee. Pediatrics branch, chinese medical doctor association. Expert consensus statement of China on the use of catheter ablation in children. *Chin J Cardiac Arrhythm.* (2017) 21:462–70.

[23] Abo-Haded HM. Radiofrequency ablation changes the quality of life of children with supraventricular tachycardias. *Arch Dis Child.* (2015) 100:754–7. 10.1136/archdischild-2014-306466 25838334

[24] Hernández-Madrid A Hocini M Chen J Potpara T Pison L Blomström-Lundqvist C. How are arrhythmias managed in the paediatric population in Europe? Results of the European heart rhythm survey. *Europace.* (2014) 16:1852–6. 10.1093/europace/euu313 25417228

[25] Desai M Kahaly O Aslam A Saifa-Bonsu J Usmani M Okabe T Comprehensive strategies to minimize radiation exposure during interventional electrophysiology procedures: state-of-the-art review. *Expert Rev Med Devices.* (2020) 17:1183–92. 10.1080/17434440.2020.1819789 32885677

[26] Purtell CS Kipp RT Eckhardt LL. Into a fluoroless future: an appraisal of fluoroscopy-free techniques in clinical cardiac electrophysiology. *Curr Cardiol Rep.* (2021) 23:28. 10.1007/s11886-021-01461-y 33655436 PMC7925460

[27] Žižek D Antolič B Prolič Kalinšek T Štublar J Kajdič N Jelenc M Intracardiac echocardiography-guided transseptal puncture for fluoroless catheter ablation of left-sided tachycardias. *J Interv Card Electrophysiol.* (2021) 61:595–602. 10.1007/s10840-020-00858-z 32860178

[28] Prolič Kalinšek T Šorli J Jan M Šinkovec M Antolič B Klemen L Conventional fluoroscopy-guided versus zero-fluoroscopy catheter ablation of supraventricular tachycardias. *BMC Cardiovasc Disord.* (2022) 22:98. 10.1186/s12872-022-02544-6 35282836 PMC8919640

[29] Kim YH Park HS Hyun MC Kim YN. Pediatric tachyarrhythmia and radiofrequency catheter ablation: results from 1993 to 2011. *Korean Circ J.* (2012) 42:735–40. 10.4070/kcj.2012.42.11.735 23236324 PMC3518706

[30] Pani A Giuseppina B Bonanno C Grazia Bongiorni M Bottoni N Brambilla R Predictors of zero X-ray ablation for supraventricular tachycardias in a nationwide multicenter experience. *Circ Arrhythm Electrophysiol.* (2018) 11:e005592. 10.1161/CIRCEP.117.005592 29874166

[31] Fadhle A Hu M Wang Y. The safety and efficacy of zero-fluoroscopy ablation versus conventional ablation in patients with supraventricular tachycardia. *Kardiol Pol.* (2020) 78:552–8. 10.33963/KP.15293 32301592

[32] Giaccardi M Mascia G Paoletti Perini A Giomi A Cartei S Milli M. Long-term outcomes after “zero X-ray” arrhythmia ablation. *J Interv Card Electrophysiol.* (2019) 54:43–8. 10.1007/s10840-018-0390-7 29948584

[33] Heidbuchel H Wittkampf FH Vano E Ernst S Schilling R Picano E Practical ways to reduce radiation dose for patients and staff during device implantations and electrophysiological procedures. *Europace.* (2014) 16:946–64. 10.1093/europace/eut409 24792380

[34] Miglioretti DL Johnson E Williams A Greenlee RT Weinmann S Solberg LI The use of computed tomography in pediatrics and the associated radiation exposure and estimated cancer risk. *JAMA Pediatr.* (2013) 167:700–7. 10.1001/jamapediatrics.2013.311 23754213 PMC3936795

[35] Roguin A Goldstein J Bar O Goldstein JA. Brain and neck tumors among physicians performing interventional procedures. *Am J Cardiol.* (2013) 111:1368–72. 10.1016/j.amjcard.2012.12.060 23419190

[36] Rogers C Bush N. Heart failure: pathophysiology, diagnosis, medical treatment guidelines, and nursing management. *Nurs Clin North Am.* (2015) 50:787–99. 10.1016/j.cnur.2015.07.012 26596665

[37] Balli S Kucuk M Epçaçan S. Transcatheter radiofrequency ablation using near-zero fluoroscopy in children with fascicular ventricular tachycardia: a single-centre experience. *Cardiol Young.* (2020) 30:779–84. 10.1017/S104795112000102X 32383414

